# RISK OF ALZHEIMER’S DISEASE AND RELATED DEMENTIA IN PATIENTS WITH SLOW PROGRESSIVE CANCERS

**DOI:** 10.1093/geroni/igad104.1295

**Published:** 2023-12-21

**Authors:** Igor Akushevich, Arseniy Yashkin, Vinit Nalawade, Julia Kravchenko

**Affiliations:** Duke University, Durham, North Carolina, United States; Duke University, Durham, North Carolina, United States; Duke University, Durham, North Carolina, United States; Duke University School of Medicine, Durham, North Carolina, United States

## Abstract

Evidence is accumulating that individuals with cancer diagnoses exhibit Alzheimer’s disease (AD) and related dementia (ADRD) risk profiles that differ from the general population of U.S. older adults. In this study we used SEER-Medicare data to compare the relative risk of AD/ADRD between individuals with slow-progressive cancers and the non-cancer general population. The study cohort included individuals age 65+ (N=2,023,054) with a primary diagnosis (1999-2017) of one of nine slow progressive cancers (breast, colorectal, prostate, uterine, kidney, ovarian, and urinary bladder cancers, as well as lymphomas and melanoma) and no clinical record of AD/ADRD prior to cancer diagnosis. This cohort was then matched by age to a comparable non-cancer population (N=1,142,641). The hazard ratios of AD/ADRD for each cancer compared to the non-cancer cohort were evaluated individually in 29 age-specific groups for each cancer type. All cancers had similar patterns of dependence for post-cancer AD/ADRD risks. We found that the presence of cancer was associated with higher risk of AD/ADRD at age at diagnosis 65-75; the relative risks decline with age at diagnoses becoming protective at advanced ages. Furthermore, for any given age at diagnosis the relative risk of AD/ADRD (i.e., cancer vs. non-cancer) also declines with time. Detailed discussion of possible causes of these effects including cancer treatment, genetic variation, possible trade-off effects, common risk and protective factors, possibly lower administration and adherence of AD/ADRD diagnostic procedures for individuals with cancer, and the roles of competing risks (first of all due to death cases) is presented.

